# Data‐driven evaluation of suitable immunogens for improved antibody selection

**DOI:** 10.1002/pro.70100

**Published:** 2025-03-21

**Authors:** Katharina Waury, Hlin Kvartsberg, Henrik Zetterberg, Kaj Blennow, Charlotte E. Teunissen, Sanne Abeln

**Affiliations:** ^1^ Department of Computer Science Vrije Universiteit Amsterdam Amsterdam The Netherlands; ^2^ AI Technology For Life, Department of Information and Computing Science, and Department of Biology Utrecht University Utrecht The Netherlands; ^3^ Department of Psychiatry and Neurochemistry Institute of Neuroscience and Physiology, The Sahlgrenska Academy at the University of Gothenburg Mölndal Sweden; ^4^ Clinical Neurochemistry Laboratory Sahlgrenska University Hospital Mölndal Sweden; ^5^ Department of Neurodegenerative Disease UCL Institute of Neurology London UK; ^6^ UK Dementia Research Institute at UCL London UK; ^7^ Hong Kong Center for Neurodegenerative Diseases Hong Kong China; ^8^ Wisconsin Alzheimer's Disease Research Center, University of Wisconsin School of Medicine and Public Health, University of Wisconsin‐Madison Madison Wisconsin USA; ^9^ Paris Brain Institute, ICM, Pitié‐Salpêtrière Hospital, Sorbonne University Paris France; ^10^ Neurodegenerative Disorder Research Center, Division of Life Sciences and Medicine, and Department of Neurology Institute on Aging and Brain Disorders, University of Science and Technology of China and First Affiliated Hospital of USTC Hefei People's Republic of China; ^11^ Neurochemistry Laboratory, Department of Clinical Chemistry Amsterdam Neuroscience, VU University Medical Center Amsterdam The Netherlands

**Keywords:** antibody, antigen, disorder, Human Protein Atlas, immunogen, protein structure

## Abstract

Antibodies are indispensable in laboratory and clinical applications due to their high specificity and affinity for protein antigens. However, selecting the right protein fragments as immunogens for antibody production remains challenging. Leveraging the Human Protein Atlas, this study systematically evaluates immunogen properties aiming to identify key factors that influence their suitability. Antibodies were classified as successful or unsuccessful based on standardized validation experiments, and the structural and functional properties of their immunogens were analyzed. Results indicated that longer immunogens often resulted in more successful but less specific antibodies. Shorter immunogens (50 residues or fewer) with disordered or unfolded regions at the N‐ or C‐terminus and long coil stretches were more likely to generate successful antibodies. Conversely, immunogens with high beta sheet content, transmembrane regions, or disulfide bridges were associated with poorer antibody performance. Post‐translational modification sites within immunogens appeared to mark beneficial regions for antibody generation. To support antibody selection, a novel R package, immunogenViewer, was developed, enabling researchers to easily apply these insights when immunogen sequences are disclosed. By providing a deeper understanding of immunogen suitability, this study promotes the development of more effective antibodies, ultimately addressing issues of reproducibility and reliability in antibody‐based research. The findings are highly relevant to the research community, as end users often lack control over the immunogen selection process in antibody production. The R package is freely available as part of Bioconductor: https://bioconductor.org/packages/release/bioc/html/immunogenViewer.html.

## 1 INTRODUCTION

Antibodies are among the most essential tools for sensitive and reliable protein detection in both laboratory and clinical settings (Ren et al., 2021; Roncador et al., 2016). Their specificity and affinity make them invaluable in many molecular biology‐based technologies for the detection and quantification of proteins, such as western blots (WBs) and immunohistochemistry (IHC) (Gao et al., 2018). These technologies are fundamental to both basic and applied scientific investigations, providing critical insights into biological processes and disease mechanisms. In the clinic, antibodies play a pivotal role in immunoassays to facilitate robust biomarker detection for diagnosis, prognosis, and patient stratification (Borrebaeck, 2017; Hansson et al., 2023).

Antibodies are typically produced through the immunization of animals with an antigen, a process that generates a robust immune response against the target protein (Laustsen et al., 2021). Polyclonal antibodies can subsequently be purified from the serum of the immunized animal. While other approaches are available, for example, hybridoma cell cultures, animal immunization offers many advantages. This technology is very well established and widespread (Chambers, 2005). Further, antibodies derived from animal immunization often outperform those produced by in vitro display technologies regarding affinity and specificity (Chambers, 2005; Laustsen et al., 2021).

Before the immunization of the animal, an essential step is the selection of the type of immunogen. The immunogen is the molecule injected into the animal to induce an immune response that leads to the production of the desired antibodies (Stills, 2012). When the aim is to produce antibodies recognizing a specific protein, several options are available regarding the immunogen. Other than the full‐length protein, using only one domain, a shorter fragment of the protein, or even a peptide can be considered (Brown et al., 2011). There are multiple reasons for choosing a protein fragment or peptide instead of a full‐length, natively folded protein. It may be challenging or impossible to produce and purify full proteins due to their size, complexity, or instability (Lee et al., 2010; Trier et al., 2012). In such cases, peptide production is more straightforward and can be easily outsourced (Lee et al., 2010). Additionally, the use of a peptide enables the generation of isoform‐ or proteoform‐specific antibodies to distinguish between closely related protein variants (Trier et al., 2019).

Using protein fragments or peptides for antibody generation also allows researchers to more precisely locate the epitope, which is the antibody recognition site on the protein. By selecting specific peptide sequences, the resulting antibodies already have fairly well‐characterized epitopes and known specificity (Trier et al., 2019). This can be very beneficial when developing a sandwich immunoassay. As, in this application, two different antibodies bind the desired protein simultaneously, it is vital that these two antibodies do not have the same or overlapping recognition site unless a homomeric protein is to be detected (Waury et al., 2022).

While offering multiple advantages, the selection of the most suitable peptide or protein fragment is a highly complex and critical task (Lee et al., 2010). Especially if the antibody is raised against a peptide but intended to detect the natively folded form of the protein, identifying the most appropriate immunogen sequence is vital to produce working antibodies (Trier et al., 2012). Brown et al. compared the performance of antibodies raised against peptides and full‐length proteins (Brown et al., 2011). In their study, anti‐peptide antibodies performed worse, especially in applications that required native protein detection, such as enzyme‐linked immunosorbent assays (ELISAs). Several potential pitfalls can compromise the efficacy of antibodies resulting from immunization with peptides or protein fragments. This includes the peptide having low surface accessibility (Grant, 2003), adopting a different structural conformation in isolation compared to its position within the native protein (Lee et al., 2010; Trier et al., 2012), and low specificity, which leads to cross‐reactivity with unwanted antigens (Lee et al., 2010).

While guidelines (Grant, 2003; Lee et al., 2010; Trier et al., 2012) exist to aid in the selection of suitable peptides or protein fragments, these suggestions are often not quantified or supported by extensive data. This lack of robust, data‐driven guidelines can lead to variability in antibody performance across different applications and hinder successful assay development. Recommendations include the selection of protein sequences with a high loop or turn content, high surface accessibility, and hydrophilicity (Grant, 2003; Lee et al., 2010; Trier et al., 2012). It has also been suggested to choose immunogens that are located within the N‐ or C‐terminus of the protein sequence, as these regions are often exposed and flexible (Grant, 2003; Lee et al., 2010; Trier et al., 2012). Note that hereinafter we solely focus on the suitability of immunogens in regard to facilitating antibody recognition. The aspect of immunogenicity, that is, the ability of a molecule to elicit an immune response, is not considered explicitly.

To address the challenges of the most suitable immunogen choice, our study aims to systematically analyze antibodies with known immunogens to understand the most optimal characteristics of these protein fragments. For this purpose, we utilized the Human Protein Atlas (HPA) (Uhlén et al., 2015), a unique and highly valuable data resource providing immunogen information for their in‐house produced polyclonal antibodies. The selection of the immunogens reported in the HPA already incorporates important factors including limited sequence similarity to the human proteome and the absence of transmembrane regions and signal peptides (Berglund, Andrade, et al., 2008; Berglund, Björling, et al., 2008). Further, antibodies are tested and validated in a highly consistent manner. Results for three types of antibody‐based technologies are contained in the atlas: (1) WBs allow protein identification based on molecular weight; (2) IHC uses immunostaining to localize proteins within tissue samples; (3) Immunocytochemistry (ICC) identifies subcellular protein localization within cells (Gao et al., 2018). Importantly, negative results are also published in the atlas (Nilsson et al., 2005; Uhlén et al., 2015). This allows us to define “successful” antibodies if they work in the majority of these three technologies, that is, WB, IHC, and ICC. Vice versa, “unsuccessful” antibodies are identified based on the majority of the technologies failing.

Identifying relevant properties for immunogen evaluation with this data‐driven approach is extremely relevant to the research community, considering that many molecular biology‐based technologies are developed using commercial antibodies where the end‐user cannot influence the choice of the immunogen sequence (Brown et al., 2011). Further, commercial antibodies are often lacking validation results across various applications (Björling & Uhlén, 2008). Choosing the most appropriate and reliable antibody for the intended application is an important step in overcoming the current reproducibility and reliability issues of research using antibody technologies due to hindered or unspecific antibody binding (Baker, 2015). Finally, to allow researchers to quickly and easily use the insights gained in this work in their antibody or immunogen selection process, we developed the novel open access R package *immunogenViewer*. This tool allows the evaluation of already available antibodies regarding the suitability of their immunogen region, if relevant validation is missing from providers. It can further support researchers during their selection of immunogens when they aim to generate novel antibodies.

## 2 RESULTS

To allow analysis of the properties of protein fragments suitable to be used as immunogens, the HPA was mined for relevant information regarding in‐house antibodies, most importantly their performance success across three different antibody‐based applications and their respective immunogens. This data allowed the annotation of antibodies as unsuccessful or successful and the comparison of their respective immunogen sequences. Annotations were generated, including AlphaFold2‐based structural features and curated annotations from the UniProt database, and compared to identify which properties are most important when selecting immunogen sequences.

### 2.1 The Human Protein Atlas as a suitable data source to evaluate immunogen suitability

**FIGURE 1:**
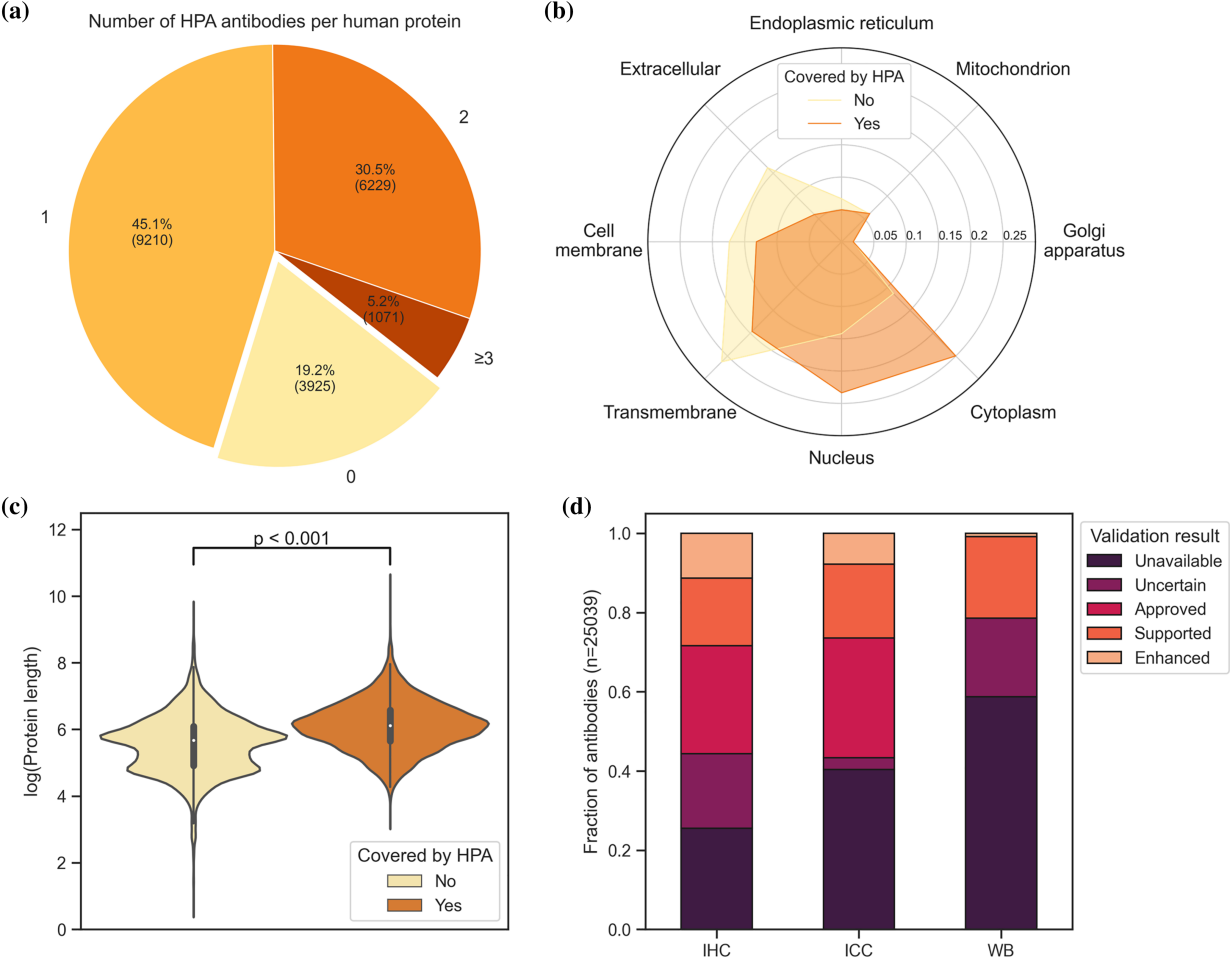
Coverage of the Human Protein Atlas. Validated HPA antibodies cover more than 80% of the human proteome. More than one‐third of the proteins are associated with multiple antibodies (a). The subset of the proteome covered by the HPA contains more proteins predicted to be located within the cytoplasm and nucleus. In comparison, proteins for which no HPA antibody is available are more likely to be at the cell membrane or in the extracellular space and contain transmembrane regions. See Table S1 for absolute protein counts per subcellular location (b). The proteome not covered by the HPA is enriched in smaller proteins with shorter sequence lengths (c). The three included antibody‐based methods (IHC, ICC, and WB) show different coverage and success rates. The most antibodies have been established in IHC; ICC is the most successful method. For less than half of the HPA antibodies, WB results are available. See Table S2 for fractions and absolute antibody counts per application (d). HPA, Human Protein Atlas;ICC, immunocytochemistry; IHC, immunohistochemistry; WB, western blot.

Data mining of the atlas led to a dataset of 23,616 antibodies. The HPA antibodies cover 80.79% of the human proteome (Figure 1a). Of the proteins of the human proteome according to UniProt, 9210 proteins (45.07%) had one antibody targeting them and 7300 proteins (35.72%) had two or more antibodies targeting them.

The part of the proteome for which no validated antibodies are available was enriched in transmembrane proteins and proteins associated with the cell membrane or the extracellular space (Figure 1b and Table S1). Visualization of the protein sequence lengths of covered and not covered proteins indicated a subset of smaller proteins for which no HPA antibodies are available (Figure 1c). A possible reason is that the immunogen selection algorithm applied for HPA antibody generation (Berglund, Björling, et al., 2008) was not able to find a protein fragment with low enough sequence identity to the human proteome in these short proteins.

According to the HPA, antibodies are categorized as “uncertain” for a tested technology (WB, IHC, or ICC), if validation experiments show undesired or differing results compared to mRNA analysis or literature. Antibodies working as intended are labeled “approved,” “supported,” or “enhanced” depending on the level of evidence and validation (Thul & Lindskog, 2018). A detailed explanation of the validation reliability scores is provided within the HPA (Human Protein Atlas, n.d.). A comparison of the antibody validation results showed that coverage is not equal across the three tested technologies (Figure 1d). IHC had the highest coverage across all HPA antibodies (74.43%), compared to ICC (59.59%) and WB (41.3%). ICC had the highest success rate across all HPA antibodies for which this technology had been tested (95.06%), compared to IHC (74.8%) and WB (52%). For more than half of the HPA antibodies, WB results were not available. Note that while more WB results are deposited in the atlas, only WB results with an associated image could be accessed in a high‐throughput manner. Full antibody counts and fractions per validation category are provided in Table S2. Based on these validation results in WB, IHC, and ICC, HPA antibodies and their respective immunogens were classified as “successful” or “unsuccessful” (see Methods in Section 4).

### 2.2 Longer immunogens increase the antibody success rate but are less specific

**FIGURE 2:**
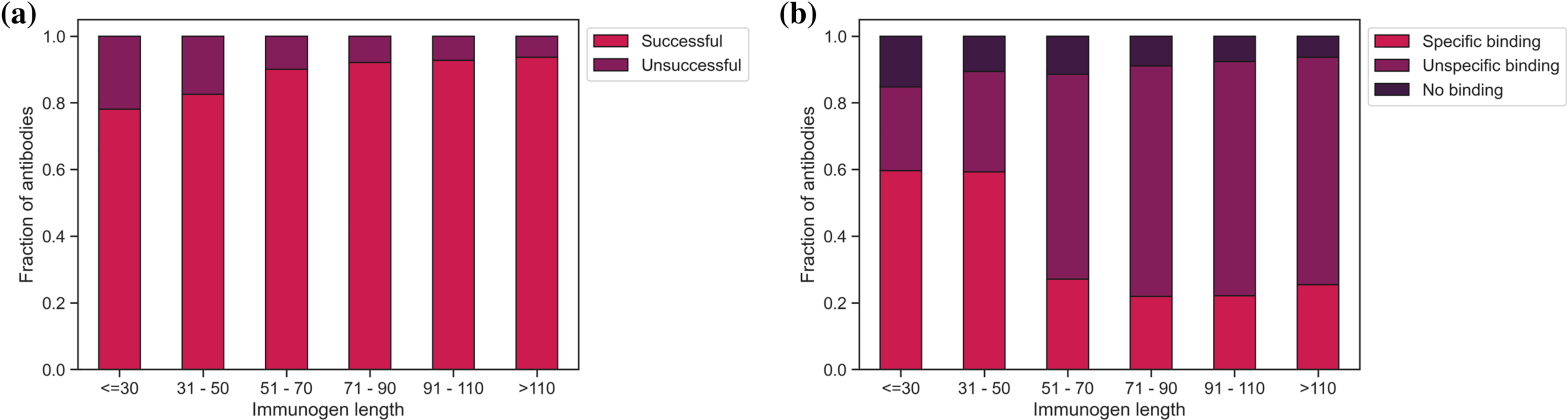
Antibody success across immunogen length. The success rate of HPA antibodies (defined by the results of validation experiments) is continuously higher with increasing length of the immunogen used for antibody generation (a). While the fraction of unsuccessful antibodies decreases in WB experiments, unspecific binding is more commonly observed in antibodies raised against longer immunogens (b). HPA, Human Protein Atlas; WB, western blot.

The included successful and unsuccessful HPA antibodies were raised against immunogens ranging in length from 19 to 202 residues. We compared the effect of increasing immunogen length both on antibody success rate and WB specificity. Longer immunogens generated successful antibodies more often than shorter immunogens. While the 707 immunogens below a length of 30 residues showed a success rate of 78.08%, this increased steadily with a growing immunogen length to a success rate of 93.68% in antibodies that were raised against immunogens of 110 residues or more (Figure 2a). On the other hand, including information on the antibody specificity highlighted that off‐target binding in WBs becomes more likely in longer immunogens (Figure 2b). Considering that HPA antibodies are polyclonal, that is, the antibodies can have differing binding regions against the utilized immunogen, these observations were to be expected. A longer immunogen provides more potential binding regions to raise working antibodies against (Lindskog et al., 2005) but might also contain regions that are similar to other protein sequences of the human proteome, leading to off‐target binding of the respective antibodies.

In the following, we focused only on immunogens comprised of 50 residues or fewer. This filter criterion still provides us with a dataset of 815 successful and 188 unsuccessful antibodies and their respective immunogens, while the antibody‐binding regions were better defined because of the limited immunogen size, allowing a more concrete analysis of immunogen suitability. This is especially relevant considering that HPA antibodies are polyclonal, and an increasing immunogen length leads to a higher number of possible epitopes and thus more diversity in the antibodies being generated (Rockberg & Uhlén, 2009). We confirmed that the amino acid type composition of the included immunogens does not differ from the excluded ones (Figure S1) to guarantee that we do not introduce bias by focusing on shorter immunogens. Further, the antibodies included in the final dataset showed comparable success rates to the full HPA antibody set across IHC (93.87%) and ICC (79.11%), while a higher fraction of these antibodies was successfully validated for WB (74.65%). A possible explanation is that many antibodies with uncertain WB results had successful validations in IHC or IHC and could thus not be classified as truly “successful” or “unsuccessful.”

### 2.3 Disordered terminus regions are confirmed as advantageous immunogen regions

Consistently mentioned properties to consider during immunogen selection are flexibility and surface accessibility (Grant, 2003; Lee et al., 2010; Trier et al., 2012). Protein disorder provides a good proxy for flexible loop regions (Teilum et al., 2009) and intrinsically disordered regions are generally known to have high solvent accessibility (Piovesan et al., 2022). We thus wished to investigate if disorder is a strong indication of immunogen suitability. Furthermore, as N‐d C‐terminal regions have been recommended specifically as suitable immunogen regions (Lee et al., 2010), we further divided our immunogens based on their position within the full protein sequence. An immunogen's position was designated as “terminus” if located at least partly within the first or last 25 residues of the protein sequence; otherwise, the position was considered “center.” These definitions separated the immunogens relatively equally into two (Table 1). Interestingly, within the HPA data, we did not observe a large difference in success rate between terminus and center immunogens (Table 1).

**TABLE 1:** Dataset compositions regarding immunogen location within the protein sequence.

Position	Number	Success rate (%)
Terminus	459	81.92
Center	538	80.48

**FIGURE 3:**
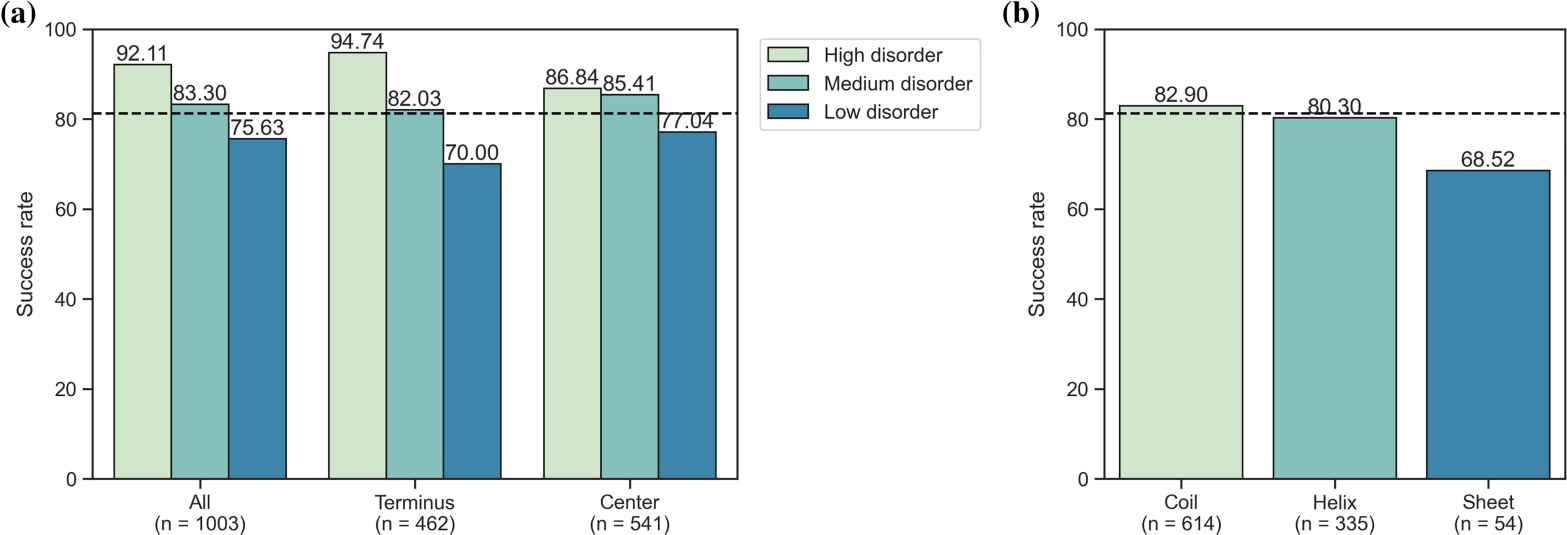
Antibody success rate in regard to disorder and secondary structure. Highly disordered immunogens are more likely to raise working antibodies. The positive effect of disorder on antibody success is more pronounced when the immunogen is located within the N‐ or C‐terminus of the protein sequence. Highly disordered immunogens within the terminus have the highest success rate. The difference in success rates is significant across all three groups (*p*_All_ < 0.001, *p*_Terminus_ < 0.001, *p*_Center_ = 0.044) (a). The most prevalent secondary structure of immunogen residues, that is, the majority secondary structure, influences antibody success rates significantly (*p* = 0.03). Coil residues are advantageous, sheet residues are the most detrimental (b). The dashed black line indicates the success rate across the full antibody set. See Table S3 for absolute antibody counts per disorder group.

We used predicted disorder scores of each immunogen residue to define 398 low, 491 medium, and 114 high disorder immunogens (see Methods in Section 4). A comparison of success rates in these disorder groups is shown in Figure 3a, while absolute antibody counts are provided in Table S3. Two trends became apparent: (1) disorder increased success rates for immunogens regardless of their positions; (2) the increased disorder content had a stronger effect on terminus immunogens. In this group, we saw an increase in the antibody success rate from low to high disorder immunogens of 35.98%, compared to an increase of only 10.65% when immunogens are located within the center.

In conclusion, high‐disorder terminal immunogens showed the highest success rates, suggesting that these regions are suitable immunogens.

### 2.4 High sheet content in immunogens is detrimental

Regions that do not form alpha helices or beta sheets, for example, connecting loop or turn regions, have been suggested to be superior immunogen choices (Trier et al., 2012). We refer to all regions that do not adopt a helix or sheet conformation as coils hereinafter. We wished to compare the impact of secondary structure on immunogen suitability as differences exist between them regarding important properties such as accessibility and stability (Kim et al., 2016; Lins et al., 2003). Residues in every immunogen were assigned as coil, helix, or sheet based on the protein structures predicted by AlphaFold2 (Varadi et al., 2024). We then identified the majority secondary structure, that is, the type that is most prevalent, for each immunogen and found significant differences between the success rates of the three classes. Immunogens dominated by coils led most often to successful antibodies (82.9%), closely followed by immunogens prevalent in helix residues (80.3%). There was a clear drop in the success rate (68.52%) when the majority of immunogen residues took a sheet conformation (Figure 3b).

Most immunogens fell into the coil majority class, while only a small number of immunogens were assigned to the sheet majority class. A comparison of the average sheet content of the full protein sequences in our dataset with the sheet content of the immunogen sequences showed that immunogens in the HPA seem to be biased towards protein fragments devoid of sheets (Figure S2a). Furthermore, in the immunogens with beta sheets being the majority secondary structure, many residues still adopted a coil or helix conformation. Immunogens in which coils or helices were most prevalent often showed that no other conformation was found (Figure S2b). These results suggest that a high content of beta sheets within immunogens is a potential obstacle for generating functioning antibodies. This issue would likely be more detrimental if no other secondary structure stretch is present that can serve as a recognition site for antibodies.

### 2.5 Structural differences between terminus‐ and center‐located immunogens

We hypothesized that immunogens located within the center are more likely to show potentially detrimental structural features compared to immunogens within the terminus regions. The investigated features included the number of distinct secondary structure elements, the number of buried residues, and the average number of intra‐chain contacts between residues. A higher number in these three features might indicate that the antibody‐binding region is less accessible and more strongly folded (Isaac & Sinha, 2015), which makes these regions less suitable than, for example, outstretched coil regions.

**FIGURE 4:**
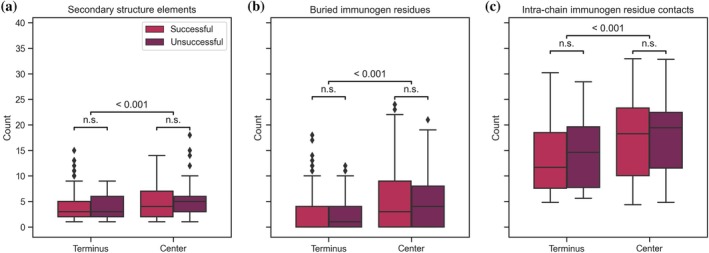
Structural differences between *terminus‐ and center‐located* immunogens. Immunogens located within the center show more potentially detrimental structural features compared to immunogens within the terminus regions, including the number of distinct secondary structure elements (a), the number of buried residues (b), and the average number of intra‐chain contacts between residues (c). Because of limited effect and dataset size, none of these differences are statistically significant between successful and unsuccessful immunogens (*p* > 0.05).

While we did see center immunogens exhibit significantly higher numbers across these three properties, we did not see any of them have a significant difference between successful and unsuccessful immunogens (Figure 4). While small disparities are apparent between the groups, the effect sizes are not large enough to be statistically significant. We could thus not confirm that a higher number of residues that are buried or have many intra‐chain contacts negatively affect immunogen suitability in our dataset.

### 2.6 Transmembrane regions, disulfide bridges, PTMs

In addition to an analysis of structural features, we were interested in identifying if functional annotations had an impact on immunogen suitability. We investigated if the presence of transmembrane regions, disulfide bridges, and post‐translational modification (PTM) sites in immunogens affected the generation of successful antibodies.

**FIGURE 5:**
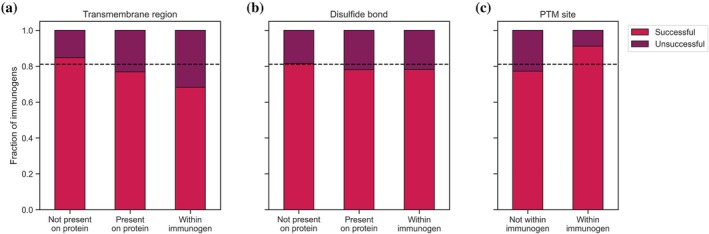
Functional annotations of immunogens. The presence of a transmembrane region within the protein antigen lowers the success rate of antibodies. The success rate is significantly lower when the immunogen used to raise antibodies overlaps with the transmembrane region (*p* < 0.001) (a). The presence of disulfide bridges lowers the success rate of antibodies, but the effect is less pronounced compared to transmembrane regions and not statistically significant (*p* = 0.687) (b). The presence of PTM sites within the immunogen is more common in successfully working antibodies (*p* < 0.001). Many PTM sites have to be accessible and are thus also available for binding. The PTM annotation does not guarantee that this site is modified when testing the HPA antibodies (c). The dashed black line indicates the success rate across the full antibody set. HPA, Human Protein Atlas; PTM, post‐translational modification.

Through a protein's incorporation into the cell membrane, part of it becomes inaccessible and thus not suitable for binding of an antibody (Berglund, Björling, et al., 2008). Thus, as is expected, the presence of a transmembrane region within the protein to be detected led to a significantly lower percentage of successful antibodies (*p* < 0.001) (Figure 5a). This observation is in line with our earlier results that proteins not covered by any HPA antibodies were enriched in transmembrane regions (Figure 1b), suggesting that for these proteins no validated antibody could be generated. The presence of transmembrane regions was most unfavorable if immunogen residues overlapped with the transmembrane region compared to immunogens that were only in the vicinity of the membrane‐bound part of the protein.

Disulfide bridges might also indicate unfavorable immunogen sequences, as these tend to be buried (Thornton, 1981), and thus the immunogen itself might be in an inaccessible position within the full protein. While we also saw a lower success rate for immunogens in which a disulfide bridge is present, the difference was not significant in this dataset compared to the presence of transmembrane regions (*p* = 0.687) (Figure 5b). Note that disulfide bridge annotations are more scarce than transmembrane regions (103 proteins with a disulfide bond vs. 318 transmembrane proteins).

Finally, we compared the effect of PTM sites within the immunogens on antibody success. Here, annotations of modified sites were more common within successful immunogens (*p* < 0.001) (Figure 5c). While PTMs have been suggested to be a potential obstacle to antibody binding if the immunogen used to raise antibodies did not contain the modification as well (Waury et al., 2022), here we saw a beneficial impact of modified sites on antibody success. It is important to consider, however, that available PTM annotations only provide information on residues that are known to have been modified. This is not direct evidence that any PTMs were actually present in the experiments done within the HPA project. As most PTM sites need to be surface accessible for a modification to be added to the relevant residue by an enzyme and for the modification to perform its function (Pang et al., 2007), PTM sites could thus be interpreted as another proxy for residue accessibility.

### 2.7 Fast and effortless immunogen evaluation with immunogenViewer

**FIGURE 6:**
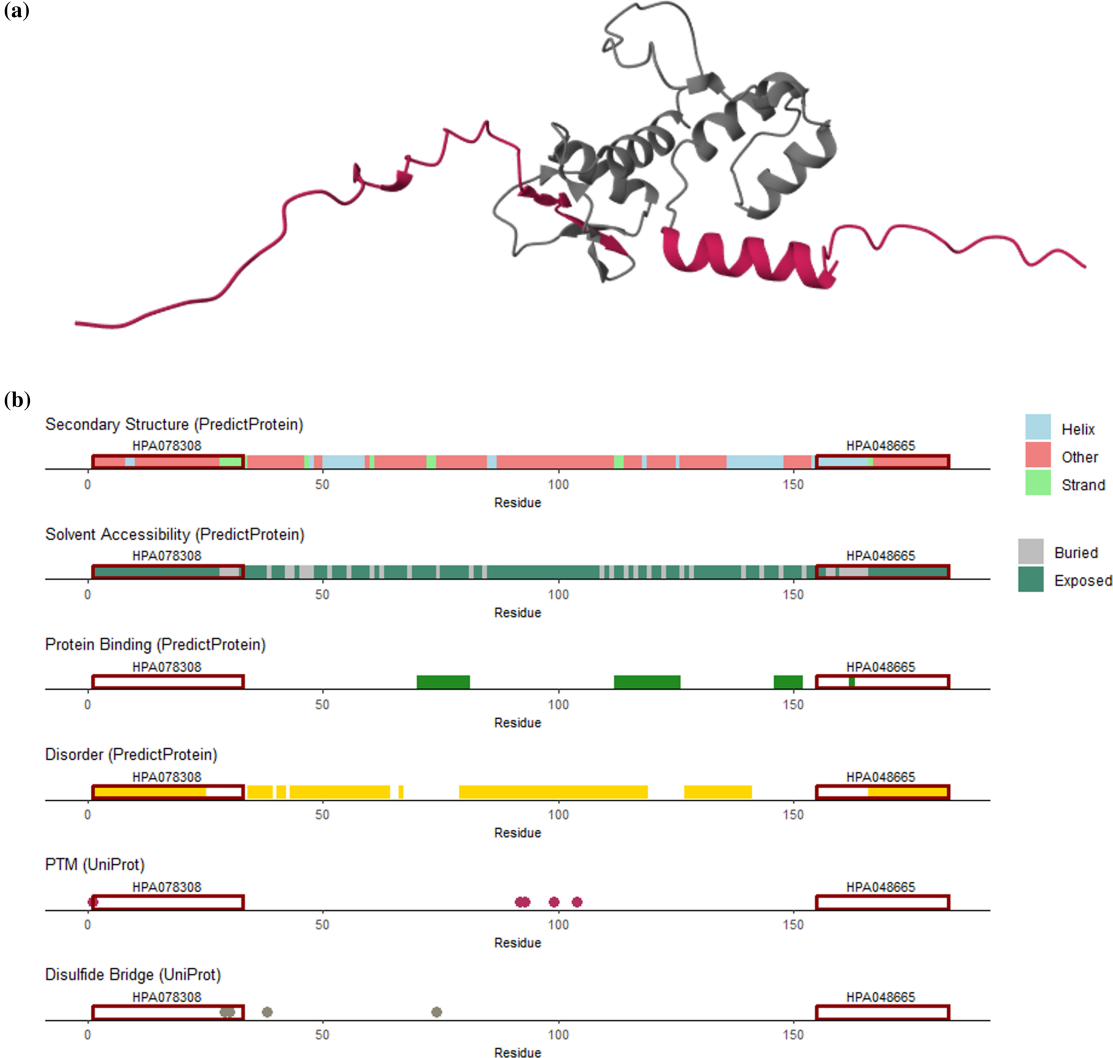
Protein visualization and immunogen evaluation with *immunogenViewer*. Protein structure of Histone deacetylase complex subunit SAP30L (UniProt ID: Q9HAJ7) according to the AlphaFold Protein Structure Database (a). Output of *immunogenViewer* visualizing several properties along the protein's sequence. Two available HPA antibodies are highlighted that can be evaluated using the package (b). HPA, Human Protein Atlas; PTM, post‐translational modification.

The comprehensive analysis of successful and unsuccessful immunogens led to a list of properties that should be considered when selecting novel or known immunogens. To facilitate an easy manner in which to evaluate the suitability of protein fragments or peptides as immunogens, we developed the R package *immunogenViewer*. This tool allows the visualization of immunogens of interest along the protein sequence and highlights relevant properties to consider, including disorder, secondary structure, surface accessibility, and PTMs. Importantly, this tool has a broad applicability as it can be used for any human protein. The package comes with a detailed manual and tutorial allowing researchers to use the package for their own needs. The package *immunogenViewer* can be accessed freely and installed easily via Bioconductor. All necessary information can be found at https://bioconductor.org/packages/release/bioc/html/immunogenViewer.html. As an example, the predicted structure of the human protein histone deacetylase complex subunit SAP30L (Figure 6a) and the visualization of its protein properties using *immunogenViewer* (Figure 6b) are shown with the immunogens of two HPA antibodies highlighted both along the structure and the sequence. Both antibodies were classified as successful based on the validation results of the HPA. The respective immunogens show a high fraction of disordered residues within the N‐ and C‐terminus, which we identified as a suitable property in this work.

## 3 DISCUSSION

Knowledge of the suitability of immunogens and their respective antibodies is strongly needed, as available guidelines on immunogen selection are scarce and not data‐driven. Our analysis aimed to address this gap, leading to the confirmation of existing recommendations such as choosing terminus and coil regions. We expanded the knowledge of optimal immunogen and antibody selection by comparing additional structural and functional features. Importantly, the relevant properties identified here can be evaluated easily and rapidly using our developed R package *immunogenViewer*.

Our study shows that properties increasing the suitability of immunogens to generate functioning antibodies include a high disorder content, especially within the terminus regions, and uninterrupted coil or helix stretches that are solvent accessible. High content of beta sheets is associated with lower success rates. In addition to the generally lower solvent accessibility of beta sheets (Lins et al., 2003), another reason for this observation could be the non‐local hydrogen bonds that are formed between beta sheets (Craveur et al., 2013). We hypothesize that protein regions with a sheet conformation within the full‐length protein may be problematic as this conformation might differ within the immunogen in isolation if the necessary hydrogen‐binding partners are not available, in addition to immunogen accessibility.

The presence of transmembrane regions and disulfide bridges decreases success rates as well. PTM sites can indicate the accessibility of a residue, explaining the higher fraction of modification sites in successful immunogens. However, the actual attachment of a modification might conceal the epitope, especially considering that HPA immunogens were produced in *Escherichia coli* (Nilsson et al., 2005) and thus might lack PTMs present in mammalian proteins, leading to differences between the immunogen and antibody target.

The HPA dataset was an invaluable resource for this study. The HPA's extensive coverage of the human proteome, the standardized methodology for antibody production and validation, and the inclusion of negative results provided a robust foundation. However, multiple drawbacks of this dataset have to be contemplated as well.

One notable issue stems from the length of the utilized immunogens of the HPA, termed protein epitope signature tags (PrESTs). With lengths often between 100 and 150 amino acids (Nilsson et al., 2005), these immunogens do not exhibit the properties of peptides and will certainly contain more than one epitope (Rockberg & Uhlén, 2009), increasing the complexity of their analysis. While we limited the dataset to immunogens of 50 amino acids or less, it is still highly likely that several epitopes exist in these immunogens. The polyclonal HPA antibodies this analysis is based on will bind different parts of the immunogen in an unknown ratio, which can impact performance immensely. This layer of uncertainty might lessen the strength of the differences observed between inadequate and suitable immunogen sequences.

Furthermore, the included antibody‐based technologies utilize denaturation and epitope retrieval steps (Nilsson et al., 2005). Those processes lead to the proteins detected changing their conformation or unfolding (Fowler et al., 2011). While this step can improve the performance of antibodies that were raised against protein fragments, it limits our insights into the antibody's performance to detect the native protein, which is the basis of immunoassays. We hypothesize that many of the trends observed in our analysis would be more pronounced if the antibodies were evaluated regarding their performance in native protein detection. In this case, the correct selection of the immunogen fragment in regard to structural properties would be even more vital (Brown et al., 2011) as buried epitopes and differences in secondary structures between immunogen and antibody target would be a larger obstacle. For example, the structural differences between successful and unsuccessful center immunogens (Figure 4) may become statistically significant. The availability of antibody performance results in technologies detecting native proteins, for example, ELISA, would significantly enhance this type of study and allow validation of this study's results in additional applications. Another issue to consider is falsely categorized antibodies and immunogens. Antibodies can be deemed “uncertain” within the HPA if the localization of proteins does not agree with the literature (Thul & Lindskog, 2018). However, this assignment might also be caused by missing literature annotations regarding the protein's expected location instead of a wrongly binding antibody. Further, WBs were defined as unspecific if bands appeared that indicated an unexpected molecular weight. This definition might again be a false negative as molecular weight can be influenced by multiple factors, for example, PTMs or dimer formation (Älgenäs et al., 2014). An unexpected molecular weight for a protein might thus again be the result of missing knowledge about this protein and its proteoforms. This ambiguity is the reason the HPA classifies these results as “uncertain.” Vice versa, the HPA usually registers the most positive performance for an antibody. For example, if a WB was tested for several samples and these show divergent results, the antibody is classified as successfully working, even if the WB shows negative results (i.e., wrong or no bands) across some of the samples. This could lead to an overestimation of the antibody's performance.

Importantly, while this study focused on protein recognition by polyclonal antibodies, the findings are likely applicable to other affinity reagents, including monoclonal antibodies, nanobodies, and emerging alternatives, such as aptamers. Challenges arising from differences between the molecules used for selection and the actual target are relevant across all affinity‐based detection methods.

Our findings underscore the importance of reporting and publishing negative or uncertain data (Björling & Uhlén, 2008). Negative results are invaluable for understanding the limitations and potential pitfalls in all research but are especially vital in improving immunogen and antibody selection. By sharing negative data, researchers can contribute to a more comprehensive and realistic understanding of antibody performance, ultimately leading to better guidelines and improved antibody development processes.

In conclusion, our analysis highlights the necessity of systematic investigations into immunogen and antibody choices. Our rigorous approach, supported by the robust data from the HPA, to define successful and unsuccessful antibodies and to evaluate a wide range of structural and functional features confirmed and expanded strongly needed recommendations for researchers working with antibody‐based technologies. To the best of our knowledge, this is the first study using the available data on HPA antibodies to gain these valuable insights for the scientific community. This study emphasizes the need for data‐driven guidelines and the importance of considering both positive and negative data in the pursuit of the generation of more effective and reliable antibodies. Future studies focusing on antibody‐based protein detection in native conditions will further enhance our understanding and refine the selection processes for immunogens and antibodies.

## 4 METHODS

### 4.1 Data collection

Information on both successful and unsuccessful antibodies, as well as their associated immunogen sequences, was required to compare relevant properties. The HPA (www.proteinatlas.org) contains standardized antibody validation results, and in the case of in‐house produced “HPA antibodies,” the utilized immunogen sequence is also available.

Relevant data of the HPA can be accessed via the XML webpages for each entry, that is, protein (e.g., http://www.proteinatlas.org/ENSG00000134057.xml). On 10 August 2023, the Ensembl IDs included in version 23.0 of the atlas were used to parse the XML pages and extract the relevant information for each protein and its associated HPA antibodies. For each antibody, the outcome of the antibody validation experiments (uncertain, approved, supported, or enhanced) in IHC, ICC, and WB was collected. For WB results, we also extracted the comments on detected bands if these were available. For 16.510 human proteins with a UniProt ID, 23,616 antibodies with a known immunogen sequence, and at least one validation were available. Note that WB results were only accessible from the XML webpages if associated with an image. Thus, a significant part of WB results could not be included in this study, explaining the large fraction of unavailable WB results within the human proteome (Figure 1d).

### 4.2 Antibody classification

The HPA provides a scale to describe the results of antibody validation experiments of IHC, ICC, and WB. For immunogen and antibody analysis, definitions were simplified: an “uncertain” result indicates that the antibody was unsuccessful. An “approved,” “supported,” or “enhanced” result indicates that the antibody was successful for this specific technology. In this study, an antibody and its immunogen are defined as “successful” if the validation results were successful for all three technologies or if the antibody was not tested for one of the technologies but was successful in the two remaining technologies. Conversely, an antibody and its immunogen are defined as “unsuccessful” if the validation failed for all three technologies or if the antibody was only tested in two of the technologies and failed for both.

The HPA provides additional information on the results of the WB experiments. Explicitly, the specificity of an antibody was evaluated by including comments on the observed bands. Thus, antibodies with WB results were further classified into specific, unspecific, and unsuccessful groups. Specific WBs detected a single band corresponding to the predicted size in kDa (±20%). Unspecific WBs showed a band differing by more than ±20% from the predicted size based on the amino acid sequence, while the band at the predicted size could either be present or absent. WBs detecting no bands were classified as “no binding.” Note that when a WB shows divergent results across different samples, the most positive outcome is registered in the atlas.

### 4.3 Immunogen analysis

To allow a trade‐off between data availability and epitope characterization, only immunogens with a length of 50 amino acids or less were included for the immunogen‐specific analysis. To exclude outliers that might skew the analysis, we also filtered for proteins of <100 or more than 2000 amino acids in length. These definitions led to 815 successful and 188 unsuccessful antibodies in our final dataset.

After the creation and filtering of the data, the final set of immunogens was analyzed to infer systematic differences between successful and unsuccessful immunogens that can guide future immunogen and antibody selection. Each immunogen was mapped to the respective full protein sequence to determine its position. HPA immunogens were classified regarding their position within the protein. “Terminus” immunogens include all immunogens that are located fully or partly within the first or last 25 residues of the full‐length protein sequence. All other immunogens are considered “center” immunogens.

### 4.4 Structural immunogen analysis

Predicted protein structures were downloaded from the AlphaFold Protein Structure Database in the format of PDB files (Varadi et al., 2024). AlphaFold models are currently the state of the art regarding protein structure prediction (Jumper et al., 2021), thus the predicted models are expected to be highly accurate. The DSSP algorithm was applied (Kabsch & Sander, 1983) to assign secondary structures (coil, helix, or sheet) and surface accessibility to each protein residue. Residues were labeled as buried if their surface accessibility within the protein structure was <7% of their potential maximum accessibility. For each immunogen residue, the number of intra‐chain contacts was calculated by counting any atom of one residue being within 8 Å of any atom of another residue. These counts were then averaged per immunogen. NetSurfP‐2.0 results of disorder prediction were utilized for disorder analysis of immunogens (Klausen et al., 2019). “High disorder” immunogens were comprised of only residues with a disorder score of at least 0.8 or higher. Vice versa, “low disorder” immunogens contained only residues with a disorder score of 0.2 or lower. All other immunogens were defined as “medium disorder.”

### 4.5 Functional immunogen analysis

Functional annotations for transmembrane residues, disulfide bridge positions, and PTM sites (i.e., annotations of modified residues, glycosylation, and lipidation) were included from UniProt (The UniProt Consortium & UniProt Consortium, 2023).

### 4.6 Subcellular localization predictions

Annotations of the predicted subcellular localization of all human proteins were available from previous work (Waury et al., 2023) and are based on the results of DeepLoc (Almagro Armenteros et al., 2017).

### 4.7 R package development

The R package *immunogenViewer* accesses available information on protein structural and functional features from the UniProt database (The UniProt Consortium & UniProt Consortium, 2023) and the PredictProtein webserver (Bernhofer et al., 2021). For extensive information and tutorials of the package, please refer to the documentation (https://bioconductor.org/packages/release/bioc/html/immunogenViewer.html). In brief, the protein features are visualized along the sequence of the protein of interest, and immunogens can be added and visualized as well.

### 4.8 Statistical testing

Testing for statistical significance was performed using the Mann–Whitney U test or Chi‐squared test. The cutoff for significance was set at 0.05.

## AUTHOR CONTRIBUTIONS

**Katharina Waury:** Conceptualization; data curation; formal analysis; investigation; methodology; software; visualization; writing – original draft. **Hlin Kvartsberg:** Conceptualization; supervision; writing – review and editing. **Henrik Zetterberg:** Writing – review and editing. **Kaj Blennow:** Writing – review and editing. **Charlotte E. Teunissen:** Writing – review and editing; funding acquisition. **Sanne Abeln:** Conceptualization; supervision; writing – review and editing.

## CONFLICT OF INTEREST STATEMENT

K. B. has served as a consultant and on advisory boards for Abbvie, AC Immune, ALZPath, AriBio, BioArctic, Biogen, Eisai, Lilly, Moleac Pte. Ltd., Neurimmune, Novartis, Ono Pharma, Prothena, Roche Diagnostics, Sanofi, and Siemens Healthineers; has served on data monitoring committees for Julius Clinical and Novartis; has given lectures, produced educational materials, and participated in educational programs for AC Immune, Biogen, Celdara Medical, Eisai, and Roche Diagnostics; and is a co‐founder of Brain Biomarker Solutions in Gothenburg AB (BBS), which is a part of the GU Ventures Incubator Program. H. Z. has served on scientific advisory boards and/or as a consultant for Abbvie, Acumen, Alector, Alzinova, ALZPath, Amylyx, Annexon, Apellis, Artery Therapeutics, AZTherapies, Cognito Therapeutics, CogRx, Denali, Eisai, LabCorp, Merry Life, Nervgen, Novo Nordisk, Optoceutics, Passage Bio, Pinteon Therapeutics, Prothena, Red Abbey Labs, reMYND, Roche, Samumed, Siemens Healthineers, Triplet Therapeutics, and Wave; has given lectures sponsored by Alzecure, BioArctic, Biogen, Cellectricon, Fujirebio, Lilly, Novo Nordisk, Roche, and WebMD; and is a co‐founder of Brain Biomarker Solutions in Gothenburg AB (BBS), which is a part of the GU Ventures Incubator Program. C. E. T. performed contract research for Acumen, ADx Neurosciences, AC‐Immune, Alamar, Aribio, Axon Neurosciences, Beckman–Coulter, BioConnect, Bioorchestra, Brainstorm Therapeutics, Celgene, Cognition Therapeutics, EIP Pharma, Eisai, Eli Lilly, Fujirebio, Grifols, Instant Nano Biosensors, Merck, Novo Nordisk, Olink, PeopleBio, Quanterix, Roche, Toyama, and Vivoryon. She is editor in chief of Alzheimer Research and Therapy and serves on editorial boards of Medidact Neurologie/Springer and Neurology: Neuroimmunology & Neuroinflammation. She had speaker contracts for Eli Lilly, Grifols, Novo Nordisk, Olink, and Roche. All declarations of interest are outside the work presented in this article.

## Supporting information


**Data S1** Supporting Information.

## Data Availability

The data that support the findings of this study are available from the corresponding author upon reasonable request.
