# Supplementary material for: Data‐driven evaluation of suitable immunogens for improved antibody selection
Source: Protein Sci. 2025 Mar 21;34(4):e70100. doi: 10.1002/pro.70100 (PMC11926642; doi:10.1002/pro.70100)
Supplement: Supplementary file 1 — Data S1 Supporting Information. [file PRO-34-e70100-s001.docx]

**Supplementary Figures**


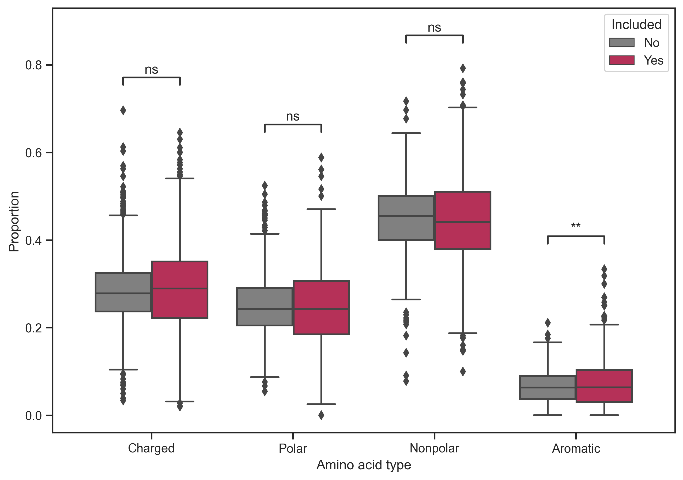


**Figure S1. Amino acid composition of included and excluded immunogens.** The fraction of amino acid types (Charged = K, R, H, D, E; Polar = S, T, N, Q, Y; Nonpolar = A, G, V, I, L, M, F, W, P; Aromatic = F, Y, W) does not significantly differ (all p > 0.05) between the immunogen sequences that were included and the ones that were excluded because of protein length (<100 or >2000 residues) or immunogen length (>50 residues).


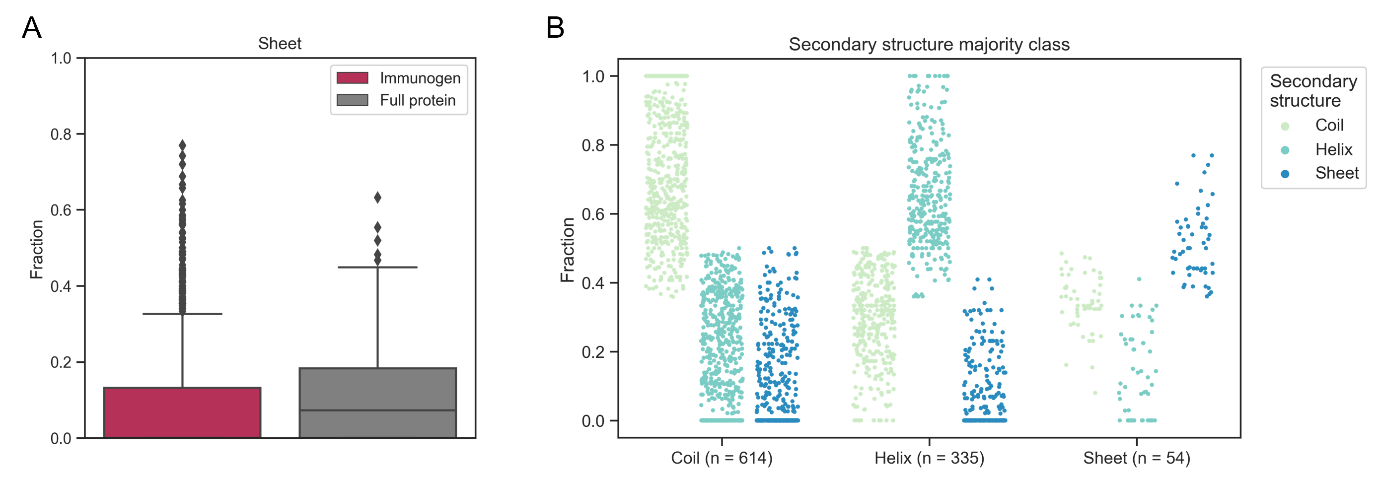


**Figure S2. Sheet content in HPA immunogens.** Compared to the full protein sequences of the HPA dataset, the selected immunogen regions are depleted of sheet residues (A). Most immunogens are dominated by coil residues. The small set of immunogens in which sheets are the majority secondary structure still contains fractions of coils and helices (B). HPA - Human Protein Atlas

**Supplementary Tables**

**Table S1. Protein numbers across predicted subcellular locations.** Distribution of proteins based on their predicted subcellular localization and their coverage by Human Protein Atlas (HPA) antibodies. ER – endoplasmic reticulum; HPA – Human Protein Atlas; TM – transmembrane

| In HPA | Cytoplasm | Nucleus | TM | Cell membrane | Extracellular | ER | Mitochondrion | Golgi apparatus |
| --- | --- | --- | --- | --- | --- | --- | --- | --- |
| Yes | 5005 | 4683 | 3929 | 2646 | 1195 | 992 | 1235 | 360 |
| No | 577 | 723 | 1336 | 884 | 824 | 339 | 310 | 92 |

**Table S2. Antibody numbers and fractions across validation categories and applications.** The fractions of the full antibody set are shown for each validation reliability category and application. Count provides the number of antibodies per reliability category while cumulative count provides the number of antibodies with this or a higher reliability score considering the order of approved < supported < enhanced. IHC – immunohistochemistry; ICC – immunocytochemistry; WB – western blot

| Validation category | IHC | | | ICC | | | WB | | |
| --- | --- | --- | --- | --- | --- | --- | --- | --- | --- |
|  | Fraction | Count | Cumulative count | Fraction | Count | Cumulative count | Fraction | Count | Cumulative count |
| Unavailable | 25.56% | 6401 | - | 40.41% | 10.118 | - | 58.7% | 14.698 | - |
| Uncertain | 18.76% | 4696 | - | 2.94% | 737 | - | 19.83% | 4964 | - |
| Approved | 27.3% | 6836 | 13.942 | 30.22% | 7566 | 14.184 | - | - | - |
| Supported | 17.06% | 4272 | 7106 | 18.65% | 4670 | 6618 | 20.6% | 5159 | 5377 |
| Enhanced | 11.32% | 2834 | 2834 | 7.78% | 1948 | 1948 | 0.87% | 218 | 218 |

**Table S3. Antibody numbers across disorder groups.** Distribution of antibodies across different disorder levels (High, Medium, Low) and their corresponding success rates. The data is further divided into terminus and center based on antibody position.

| Position | Disorder | Count | Success rate |
| --- | --- | --- | --- |
| All | High | 114 | 92.11% |
|  | Medium | 491 | 83.3% |
|  | Low | 398 | 75.63% |
| Terminus | High | 76 | 94.74% |
|  | Medium | 306 | 82.03% |
|  | Low | 80 | 70% |
| Center | High | 38 | 86.84% |
|  | Medium | 185 | 85.41% |
|  | Low | 318 | 77.04% |
